# Validity and psychometric characteristics of the Duruöz Hand Index (DHI) in patients with systemic sclerosis

**DOI:** 10.1007/s00296-025-05829-z

**Published:** 2025-03-15

**Authors:** Mehmet Tuncay Duruöz, Nuran Öz, Yusuf Karabulut, Didem Erdem Gürsoy, Halise Hande Gezer, Sevtap Acer Kasman

**Affiliations:** 1 Physical Medicine and Rehabilitation Department, Rheumatology Division, Marmara University School of Medicine, Istanbul, Türkiye; 2 Internal Medicine Department, Rheumatology Division, Yıldırım Doruk Hospital, Bursa, Türkiye; 3 Physical Medicine and Rehabilitation Department, Rheumatology Division, Prof. Dr Cemil Taşcioğlu City Hospital, Istanbul, Türkiye

**Keywords:** Systemic sclerosis, Duruöz hand index, Hand function, Validation

## Abstract

Objective: The Duruöz Hand Index (DHI) is a self-report questionnaire originally developed to assess hand function in rheumatoid arthritis patients and validated for various rheumatic conditions. The aim of the study is to evaluate the validity and psychometric features of the DHI in patients with systemic sclerosis (SSc). Methods: SSc patients diagnosed using EULAR/ACR 2013 criteria were included in study. Hand functionality was assessed using the DHI, Hand Functional Index (HFI) and visual analog scales (VAS) for disability and handicap. Overall disability and quality of life were measured using the Health Assessment Questionnaire (HAQ) and Short Form-36 (SF-36). Reliability (Cronbach’s alpha and ICC) and validity (face, content, convergent and divergent) were also analyzed and correlations with other measures assessed for construct validity of the DHI were examined. Results: Seventy-three patients were included in the study, 78.1% were female. The baseline mean DHI score was 31.2 (SD: 20.2). Completion and calculation of the questionnaire were easy and took 5 min and 30 s, respectively. The Cronbach’s alpha coefficient for internal consistency was 0.973 and the ICC for test-retest reliability was 0.993 (95%CI 0.981–0.997), suggesting that the DHI has high internal consistency and a high degree of reliability. Cognitive debriefing showed that the DHI is clear, understandable, relevant, and covers many domains of daily life, indicating good face and content validity. The DHI demonstrated good to moderate correlations with functional measures indicating convergent validity and moderate to non-significant correlations with non-functional parameters that supported divergent validity. Cronbach’s alpha was 0.973, indicating excellent internal consistency. Conclusion: In SSc patients, specific tools for assessing hand function are lacking. The DHI is a practical, reliable and valid measurement tool for both clinical assessment and research in this disease affecting skin, tendons, subcutaneous tissue and arthritis.

**Supplementary Information:**

The online version contains supplementary material available at 10.1007/s00296-025-05829-z.

## Introduction

Systemic sclerosis (SSc) is a complex autoimmune disease that profoundly affects multiple organ systems. Its hallmark features include microvascular damage, progressive fibrosis, and immune dysregulation, culminating in significant morbidity and mortality [1]. In advanced stages, SSc often leads to obliterative vasculopathy and extensive fibrosis in internal organs, contributing to severe pulmonary and cardiac complications. These complications underline the high prevalence of the disease in women, with a female-to-male ratio of approximately 4:1, and a typical onset between 30 and 60 years of age [2]. Despite advances in treatment, SSc remains a disease with high disease burden, necessitating reliable outcome measures to monitor disease impact and progression.

The disease trajectory of SSc has a notable impact on hand function. Early-stage vascular involvement results in Raynaud’s phenomenon (RF) and ischemic changes, while later stages are characterized by skin thickening, ulceration, and necrosis, which can lead to contractures and further restrict hand movement. Such impairments not only exacerbate physical disability but also diminish quality of life by interfering with daily activities such as dressing, personal hygiene, and household tasks. Hand dysfunction, compounded by symptoms like joint stiffness, fatigue, and pain, significantly reduces functional independence in patients [3] Given that hand involvement is almost universal in SSc, useful assessment tools are crucial to capture the extent of disability and guide therapeutic interventions.

The Duruöz Hand Index (DHI), a self-report questionnaire that assesses 18 daily activities covering tasks such as kitchen work, dressing and office-related functions, is both practical and effective. The assessment requires minimal time and no specialized equipment. Initially developed for rheumatoid arthritis (RA), the index has proven reliable in a variety of rheumatic and non-rheumatic conditions. In SSc, it is a valuable tool used in studies to assess hand-related disability. Its clinical relevance is further supported by studies to assess and improve hand function in SSc [4–6]. Unlike more generalized disability assessments, the DHI specifically evaluates fine motor skills, grip strength, and dexterity, making it a potentially more sensitive measure for SSc-related functional impairment. By focusing on hand-specific activities, the DHI offers a targeted approach to understanding functional limitations in SSc, making it an important tool for clinical and research applications [7].

This study aims to validate the psychometric properties of the DHI in patients with SSc, emphasizing its reliability, usability, and relevance in capturing hand dysfunction within this patient population.

## Materials and methods

### Study design and participants

This cross-sectional study included patients aged 18 to 65 years who were diagnosed with limited cutaneous SSc or diffuse cutaneous SSc based on the EULAR/ACR 2013 classification criteria [8]. Exclusion criteria encompassed neuropathic pain, neurological disorders affecting the upper extremities, malignancy, recent hand trauma or surgery (within 90 days), upper extremity amputation, pregnancy, or severe psychiatric conditions. Ethical approval was granted by the Marmara University Faculty of Medicine Research Ethics Committee and informed consent was obtained from all participants.

### Data collection

The study followed a two-stage cross-sectional design. In the first stage, a rheumatologist recorded patient demographics, including age, gender, disease duration, SSc subtype and hand dominance. Clinical data, such as the number of tender and swollen joints, presence of active digital ulcers, pitting scars, and digital amputations, were also documented. In the second phase, patients also completed questionnaires assessing hand pain, morning stiffness, RF and disability for the previous week by use of a Visual Analog Scale (VAS).

### Assessment tools

DHI, Hand Functional Index (HFI), and Health Assessment Questionnaire (HAQ) were used for functional assessments of the hand [9–11]. Short Form-36 (SF36) was used to assess quality of life [3]. The European Scleroderma Study Group (EScSG) activity index [12] and VAS physician’s opinion of activity were used to estimate disease activity. Modified Rodnan skin score (mRSS) [13], Pittsburgh sleep quality index (PSQI) [14], and CRP levels were also recorded.

### Functional indexes

The DHI is a validated tool designed to assess functional impairment due to hand activity limitations. Initially developed for rheumatoid arthritis, it has since been adapted for use in other conditions, including diabetes, cerebral palsy, carpal tunnel syndrome, tetraplegia, systemic sclerosis, and stroke [11]. The questionnaire consists of 18 items that measure manual dexterity in various daily tasks, such as self-care, household activities, office-related tasks, dressing, and general movements. Each item is scored on a Likert scale from 0 (no difficulty) to 5 (unable to perform), reflecting the individual’s functional challenges over the past week. The total score ranges from 0 to 90, with higher scores indicating greater disability [7]. The full version of the DHI is provided as Supplementary Material (Supplementary 1).

Even though the HAQ was established initially to assess the functional ability of individuals diagnosed with RA, it has been used with confidence in a variety of diseases. The questionnaire consists of eight subgroups assessing activities of daily living: dressing, getting up, hygiene, eating, walking, reaching, grasping and other activities. Questions in each subscale include 2 or 3 questions and responses are categorised as 0 (cannot be done with no difficulty), 1 (can be done with some difficulty), 2 (can be done with great difficulty) and 3 (cannot be done). The final HAQ score may vary between 0 and 3 [10].

The HFI is comprised of the initial 9 questions of the Keitel Functional Test. It serves as a hand scale assessing finger and wrist movements for the upper extremity, generating a total score between 4 and 42. The higher scores indicating poorer function [11].

A comprehensive quality of life questionnaire, the SF-36 measures eight main domains: physical functioning, physical health-related role limitations, pain, general health, vitality, social functioning, emotional health-related role limitations, and mental health [3].

Internal consistency of the DHI was assessed using Cronbach’s alpha coefficient, while test-retest reliability was assessed using the intraclass correlation coefficient (ICC). To perform the test-retest analysis, 20 patients completed the DHI a second time one week later to assess score consistency.

### Reliability analyses of DHI in SSc

To assess the reliability of the DHI in SSc patients, both internal consistency and test-retest reliability were examined. Internal consistency was evaluated by calculating Cronbach’s alpha based on the overall DHI scores. For test-retest reliability, patients completed the DHI again after a one-week interval, and the stability of their responses was measured using the intraclass ICC.

### Validity analyses of DHI in SSc

The validity of the DHI in SSc was examined through assessments of face, content, and construct validity. Face validity was evaluated using cognitive debriefing interviews with participants to ensure that the questionnaire was clear, easily understood, and appropriate for patients. Content validity was determined through expert physician reviews, assessing whether the items comprehensively covered the functional challenges faced by individuals with SSc. Construct validity was explored through convergent and divergent validity analyses. Convergent validity was demonstrated by strong correlations between DHI scores and established functional measures such as HFI, HAQ, and VAS for disability. Divergent validity was supported by weak or non-significant correlations between DHI scores and variables unrelated to functional impairment.

### Statistical analysis

Statistical analyses were performed using SPSS 24.0. Quantitative variables are presented as mean ± standard deviation, while categorical variables are expressed as frequencies (percentages) or median with interquartile range. The reliability of the DHI was evaluated using Cronbach’s alpha to assess internal consistency and the intraclass correlation coefficient (ICC) to determine test-retest reliability. Spearman’s correlation coefficient was used to analyze relationships among quantitative variables. Correlations were classified as strong if rho exceeded 0.6, moderate if between 0.3 and 0.6, and weak or negligible if below 0.3. A *p*-value of less than 0.05 was considered statistically significant.

## Results

### Study population

A total of 73 patients with a mean age of 48.5 years (SD: 12.7) were included in the study, of whom 57 (78%) were female. The average disease duration was 9.9 years (SD: 9.0), and almost all patients were receiving treatment. The baseline mean DHI score was 31.2 (SD: 20.2), with a median of 8.5 (IQR: 21). Detailed demographic and disease characteristics are provided in Table 1.

**Table 1:** Demographic and clinical characteristics of patients

Age, years	48,5 (SD 12,7)
Disease duration, years	9.9 (SD 9)
Subgroups in SSc, n (%)	
Limited cutaneous SSc Diffuse cutaneous SSc SSc limited to skin Prescleroderma Overlap autoimmune disease	28 (38.4%) 33 (45.2%) 8 (11%) 1 (1.4%) 3 (4.1%)
Hand dominancy	
Right, n (%)	67 (91.8%)
Clinical manifestations	
Raynaud’s Digital ulcers Digital amputation Telengiectasia Calcinosis cutis Synovitis Flexion contractures Tendon friction rubs Arrhythmia Interstitial lung disease Pulmonary hypertension	66 (90,4%) 29 (39,7%) 8 (11,0%) 70 (95,9%) 13 (17,8%) 25 (34,2%) 10 (13,7%) 6 (8,2%) 5 (6,8%) 29 (39,7%) 14 (19,2%)
CRP, mg/L	3.16 (IQR 4.67)
mRSS	18 (IQR 20)
EScSG	2,5 (IQR 2,63)
HAQ	1,72 (SD 9.84) (IQR 4,31)
SF-36	
Physical Function Emotional Role Emotional Well-being Social Functioning Pain Physical Role Limitation Energy General Health Health Change	50 (IQR 46) 33,3 (IQR 100) 50 (IQR 24) 50 (IQR 25) 45 (IQR 35) 25 (IQR 75) 50 (IQR 30) 32,5 (IQR 25) 50 (IQR 25)
VAS-p pain	50 (IQR 30)
VAS-p raynoud	50 (IQR 20)
VAS-p disabilite	50 (IQR 33)
VAS-p handicup	50 (IQR 40)
Pittsburgh Sleep Scale	9 (IQR 8)

Abrivatttion: SD, standar derivation;

Abbreviation: CRP C-reactive protein, DHI Duruöz Hand Index, EScSG European Scleroderma Study Group, HAQ Health Assessment Questionnaire, HFI Hand Functional Index, mRSS Modified Rodnan Skin Score, PSQI Pittsburgh Sleep Quality Index, SF-36 Short Form-36 Health Survey, SJC Swollen Joint Count, TJC Tender Joint Count, VAS Visual Analog Scale

### Validity assessments

The face validity is verified through the cognitive debriefing interviews with the patients. Debriefing showed that the DHI was clear, understandable and relevant. The DHI is easy to complete and easy to estimate, taking 6 min respectively. Moreover, the DHI demonstrated strong content validity since its items proved suitable for assessing multi-domain hand disability in patients with SSc. In assessing content validity, reviews confirmed that the DHI adequately covers multiple dimensions of hand dysfunction in SSc, including dexterity, grip strength and fine motor control.

In Convergent Validity, the DHI showed strong correlations with other functional measures, confirming its ability to assess hand-related disability in SSc. The highest correlation was observed with the HFI (rho: 0.970, *p* < 0.001). Moderate correlations were found with VAS-disability (rho: 0.524, *p* < 0.001), VAS-disability (rho: 0.567, *p* < 0.001) and the HAQ (rho: 0.395, *p* < 0.01), supporting convergent validity (Table 2).

**Table 2:** Spearman’s correlation coefficients of duruöz hand index with the other parameters for convergent and divergent validity

Functional Parameters (Convergent)	Rho	Non-functional Parameters (Divergent)	Rho
HFI	0.970^**^	Age	-0.124
VAS-p Disability	0.524^**^	Disease duration	0.347^*^
VAS-p Handicap	0.567^**^	CRP	0.246^*^
HAQ	0.395^*^	SJC	0.273^*^
		TJC	0.464^**^
		mRSS	0.495^**^
		EScSG	0.535^**^
		VAS-Raynoud	0.419^**^
		VAS-Hand pain	0.512^**^
		VAS-Doctor’s activity opinion	0.484^**^
		SF36 Physical functioning	-0.338^*^
		SF36 Physical role limitations	-0.525^**^
		SF36 Emotional role limitations	-0.375^*^
		SF36 Vitality	-0.164
		SF36 Emotional well-being	-0.085
		SF36 Social functioning	-0.336^*^
		SF36 Pain	-0.259^*^
		PSQI	0.305^*^

** *p* < 0.001, *p: 0.001–0.049

Abbreviation: CRP C-reactive protein, DHI Duruöz Hand Index, EScSG European Scleroderma Study Group, HAQ Health Assessment Questionnaire, HFI Hand Functional Index, mRSS Modified Rodnan Skin Score, PSQI Pittsburgh Sleep Quality Index, SF-36 Short Form-36 Health Survey, SJC Swollen Joint Count, TJC Tender Joint Count, VAS Visual Analog Scale

In Divergent Validity, the DHI also showed weak or insignificant correlations with non-functional measures such as age (rho: -0.124, *p* = 0.28), disease duration (rho: 0.347, *p* = 0.06) and CRP levels (rho: 0.246, *p* = 0.09), confirming that it specifically measures functional disability rather than unrelated disease parameters. In addition, no or moderate correlations were observed with swollen joint count and tender joint count (rho: 0.273–0.464), VAS pain, mRSS (rho: 0.419) and the EScSG activity index (rho: 0.535). SF-36, VAS activity, VAS Raynaud’s and PSQI (rho: 0.305–0.512) further support the differential validity of the DHI (Table 2).Convergent and divergent validities results show us the construct validity of the DHI in SSc.

The Cronbach’s alpha coefficient for the DHI was 0.973, indicating excellent internal consistency, meaning that the questionnaire items are highly interrelated and measure the same construct.

Twenty patients completed the DHI a second time after one week to assess test-retest reliability. There was no significant difference in scores between the two time points (Wilcoxon test: *p* = 0.108), and the ICC was 0.993 (95% CI: 0.981–0.997, *p* < 0.001). This confirms excellent test-retest reliability, meaning that the DHI produces consistent results over time.

## Discussion

This study aimed to evaluate the reliability and validity of the DHI for SSc patients in our community. The findings indicated excellent reliability and construct validity. Musculoskeletal and skin involvement arising from SSc can result in notable deformities and functional loss. There are specific and nonspecific tools designed for assessing hand function in SSc patients. Evaluating hand involvement in SSc necessitates a combination of outcome measures. Patient-reported outcomes (PRO) measures are commonly employed and represent an appropriate method for assessing patients’ perceptions regarding the impact of their disease. The DHI, being a PRO, is extensively utilized across various diseases that can lead to hand dysfunction. These tools, facilitating patients in assessing various aspects of their diseases, must possess validity, reliability, and applicability [15].

Hand involvement is common in SSc and significantly affects quality of life. It encompasses joint, skin and tendon complications, including skin sclerosis, RF, digital ulcers, myositis and sclerosing tenosynovitis. Synovitis has been reported in 15–47.5% of patients with SSc. While synovial involvement was found in half of the patients (47.5%) in a scleroderma cohort of 1483 patients in Germany, the prevalence of synovitis was found to be 15% in an analysis of the European Scleroderma Trials and Research Group (EUSTAR) database of 7286 SSc patients [16, 17]. In SSc, enthesitis and involvement of the synovial-entheses complex have been described and finger flexion contractures are an important cause of disability. Similar to RA and PSA, tendon involvement is present, but tendon involvement is less inflammatory and more fibrotic (sclerosing tenosynovitis) [18]. Although calcinosis is observed in 20–40% of patients with SSc, it occurs most commonly in the hands and especially in the thumb [19]. SSc may involve all compartments of the hand including joints, skin, tendons and enthesal region and may cause a serious impairment in hand functions.

DHI has demonstrated significant utility in evaluating hand function impairments in SSc by quantifying functional limitations in daily activities. In the study by Akatay et al. performance-based hand functions were assessed in SSc patients using the Sollerman Hand Function Test (SHFT), and a strong correlation was found between SHFT and DHI scores [4]. This finding highlights that the DHI effectively captures patient-reported functional disability, aligning well with objective hand function assessments. The study also confirmed that DHI is particularly useful in evaluating fine motor skills and dexterity, which are frequently impaired in SSc due to joint stiffness, skin thickening, and vascular complications.Moreover, in a randomized controlled trial evaluating the efficacy of home self-administered isometric hand exercises for 8 weeks in patients with SSc, they investigated the therapeutic applicability of the DHI. Their findings showed that patients who followed the hand exercises showed significant improvements in DHI scores, reinforcing the role of the DHI in monitoring treatment response and guiding rehabilitation strategies [5]. The study highlights the sensitivity of the DHI to functional changes, making it a valuable tool for monitoring disease progression and intervention outcomes in both clinical and research settings. Additionally, the study by Gündüz et al. provided further evidence for DHI’s clinical relevance, revealing a positive correlation between serum SCUBE-2 levels and DHI scores.This suggests that DHI not only reflects functional impairment but may also be associated with underlying pathophysiological mechanisms such as fibrosis, vascular dysfunction, and inflammation in SSc [6]. Together, these findings emphasize the DHI’s role as a sensitive and practical tool for assessing hand function in SSc. It facilitates comprehensive patient evaluation, aids in therapeutic monitoring, and potentially links biomarkers to functional impairment, thereby enhancing the clinical management and rehabilitation of SSc-related hand disability.

To assess hand function in SSc patients, specific tools are available. Evaluating hand involvement in SSc necessitates a combination of outcome measures. Hand involvement assessments that have previously been reported to be reliable and valid for people with SSc include the Arthritis Hand Function Test [20], which measures hand strength and dexterity, and the Scleroderma Hand Mobility Test [21], which evaluates hand mobility. In our study, there is a strong correlation between both hand function assessment tests [22].

The DHI, initially developed for assessing hand function in RA patients, has undergone subsequent validation in various conditions. It has been validated not only in inflammatory conditions but also in non-inflammatory ones where the assessment of hand function is essential. These conditions include cerebral palsy, psoriatic arthritis, carpal tunnel syndrome, stroke and tendon injuries [9, 23–26].

To evaluate the internal consistency and test-retest reliability of the DHI, Cronbach’s alpha coefficient and ICC were used. The DHI demonstrated high reliability in the SSc case, achieving an ICC score of 0.973. Consistent with findings from previous studies applying the DHI in various patient populations. ICC values of 0.96, 0.97 and 0.97 were found in psoriatic arthritis, carpal tunnel syndrome and stroke patients, respectively [23–25]. The reliability of the DHI test-retest is quite high (ICC = 0.993) and is consistent with similar studies in patients with carpal tunnel syndrome and cerebral palsy [7, 21]. These results confirm the reliability and reproducibility of the DHI, consistent with observations in different populations [9, 23–26]. Furthermore, the DHI offers advantages such as ease of use, efficient time utilization, comprehensiveness, and financial efficiency. Notably, the high ICC observed in this study confirms the DHI’s capacity to deliver stable and consistent results, even in the presence of SSc-related variability in disease presentation. Its multidimensional approach to assessing daily living activities also provides a comprehensive overview of functional impairments, which is critical in diseases like SSc where hand function is multifactorially affected.

The convergence validity of DHI scores in SSc patients was assessed by examining their correlation with other functional parameters. Notably, DHI scores showed a high correlation with HFI and a moderate correlation with VAS disability, vas handicap, and HAQ. Among these parameters, HFI demonstrated the strongest association with DHI. Originally designed to evaluate physical function in RA, the HAQ has found widespread use in various inflammatory arthritides. The expected strong correlation between DHI and HAQ, an assessment of functional ability, was observed. Interestingly, some elements in the HAQ were noted not to pose significant problems for the functionality of individuals with SSc. While the widely-used HAQ evaluates both upper and lower extremities, the DHI uniquely concentrates on hand functi ve spon. Therefore, the distinct advantage of DHI lies in its specificity to hand function. This indicates a functional and specific promise of DHI.

When evaluating the validity of divergent constructs, the DHI demonstrated no correlation with dysfunctional parameters, including age, disease duration, CRP and PSQI thereby supporting discriminant validity. However, a weak to moderate correlation was observed with disease activity parameters such as tender joint count, swollen joint count, EScSG, and mRSS. In diseases like SSc and PsA, where joint and skin involvement may coexist, hand function impairment could potentially develop independently of disease activity. The results reveal that the DHI shows a moderate correlation with the SF-36 and a link with general health-related quality of life measures. However, a stronger correlation was observed with specific functional subgroups, emphasising its potential utility in more precisely capturing aspects of physical functioning and activity limitations. Interestingly, no significant correlations were found with other measured variables, which may underline its specificity to functional domains rather than broader systemic or psychosocial factors.This suggests that it is a functionally specific scale.

As SSc is a complex, multi-system disease with significant morbidity, tools like the DHI play a vital role in optimizing patient outcomes. DHI emerges as a valuable and practical tool for assessing hand function in SSc. With its excellent reliability, validity, and ease of use, the DHI offers clinicians an efficient and patient-centered method for evaluating the impact of SSc-related hand impairments on daily activities. It facilitates use, patient follow-up and adherence to the core parameter set for SSc.

## Conclusion

Overall, the study confirms that the DHIis a valid and reliable tool for assessing hand function in patients with SSc. With excellent internal consistency, test-retest reliability and construct validity, the DHI effectively detects functional impairments associated with SSc. Its ease of use and strong correlations with other functional measures make it a practical option for both clinical and research settings. Given the significant impact of SSc on hand function, the DHI serves as a valuable tool for assessing disability and monitoring disease progression.

## Electronic supplementary material

Below is the link to the electronic supplementary material.

Supplementary Material 1

Supplementary Material 2

Supplementary Material 3

Supplementary Material 4

Supplementary Material 5

Supplementary Material 6

Supplementary Material 7

Supplementary Material 8

## Data Availability

Data supporting the findings of this study are available from the corresponding author upon reasonable request. Data sharing complies with institutional and ethical guidelines.

## Declarations

### Note on related congress abstracts and publication

Accepted at the EULAR 2023 European Congress of Rheumatology. Published text information: M.T. Duruöz, Y. Karabulut, N. Öz, D. Erdem Gürsoy, H.H. Gezer, S. Acer Kasman. AB0834 VALIDITY AND PSYCHOMETRIC CHARACTERISTICS OF THE DURUÖZ HAND INDEX IN TURKISH PATIENTS WITH SYSTEMIC SCLEROSIS. Annals of the Rheumatic Diseases, Volume 82, Supplement 1,2023, Pages 1629–1630, ISSN 0003-4967, 10.1136/annrheumdis-2023-eular.5195.
