# Correction: Validity and psychometric characteristics of the Duruöz Hand Index (DHI) in patients with systemic sclerosis

**DOI:** 10.1007/s00296-025-05844-0

**Published:** 2025-04-18

**Authors:** Mehmet Tuncay Duruöz, Nuran Öz, Yusuf Karabulut, Didem Erdem Gürsoy, Halise Hande Gezer, Sevtap Acer Kasman

**Affiliations:** 1 Physical Medicine and Rehabilitation Department, Rheumatology Division, Marmara University School of Medicine, Istanbul, Turkey; 2 Internal Medicine Department, Rheumatology Division, Yıldırım Doruk Hospital, Bursa, Turkey; 3 Physical Medicine and Rehabilitation Department, Rheumatology Division, Prof. Dr Cemil Taşcioğlu City Hospital, Istanbul, Turkey

**Correction: Rheumatology International (2025) 45:75** 10.1007/s00296-025-05829-z

In this, article the title was incorrectly given as **“Validity and psychometric characteristics of the Duruöz Hand Index (DHI) with systemic sclerosis”** but should have been **“Validity and psychometric characteristics of the Duruöz Hand Index (DHI) in patients with systemic sclerosis”.**

The original article has been updated.

