# Supplementary material for: Validity and psychometric characteristics of the Duruöz Hand Index (DHI) in patients with systemic sclerosis
Source: Rheumatol Int. 2025 Mar 15;45(4):75. doi: 10.1007/s00296-025-05829-z (PMC11910436; doi:10.1007/s00296-025-05829-z)
Supplement: Supplementary file 1 — Supplementary Material 1 [file 296_2025_5829_MOESM1_ESM.pdf]

Duruöz Hand Index (DHI)

**Answers to the questions:**	**Score**
C1 - In the kitchen	
**1** Can you hold a bowl?	
**2** Can you seize a full bottle and raise it?	
**3** Can you hold a plate full of food?	
**4** Can you pour liquid from a bottle into a glass?	
**5** Can you unscrew the lid from a jar opened before?	
**6** Can you cut meat with a knife?	
**7** Can you prick things well with a fork?	
**8** Can you peel fruit?	
C2 - Dressing	
**9** Can you button your shirt?	
**10** Can you open and close a zipper?	
C3 - Hygiene	
**11** Can you squeeze a new tube of toothpaste?	
**12** Can you hold a toothbrush efficiently?	
C4 - In The Office	
**13** Can you write a short sentence with a pencil or ordinary pen?	
**14** Can you write a letter with a pencil or ordinary pen?	
C5 - Other	
**15** Can you turn around door knob?	
**16** Can you cut a piece of paper with scissors?	
**17** Can you pick up coins from a table top?	
**18** Can you turn a key in a lock	
**Total**	

0 : Yes, without difficulty
1 : Yes, with a little difficulty
2 : Yes, with some difficulty
3 : Yes, with much difficulty
4 : Nearly impossible to do
5 : Impossible

Patient last name: . . . . Date of birth: . . . . / . . . . / . . . .
Patient first name: . . . . Date: . . . . / . . . . / . . . .
