# Supplementary material for: Validity and psychometric characteristics of the Duruöz Hand Index (DHI) in patients with systemic sclerosis
Source: Rheumatol Int. 2025 Mar 15;45(4):75. doi: 10.1007/s00296-025-05829-z (PMC11910436; doi:10.1007/s00296-025-05829-z)
Supplement: Supplementary file 2 — Supplementary Material 2 [file 296_2025_5829_MOESM2_ESM.docx]

**Authorship and Originality Disclaimer**

We, the authors, hereby declare that this manuscript is our original work and has not been published or submitted for publication elsewhere in whole or in part. All text, data, figures, and other content are original, and no portion of this work has been duplicated from previously published sources.

Additionally, we confirm that we have adhered to ethical research and publication standards, and no part of this manuscript infringes on any copyright or intellectual property rights. If required, we are willing to provide any supporting documentation to verify the originality of this work.

By submitting this manuscript, we acknowledge and accept the journal’s policies regarding originality, duplication, and ethical publication standards.
